# Attitudes and Perceptions of University Students in Healthcare Settings towards Vaccines and Vaccinations Strategies during the COVID-19 Pandemic Period in Italy

**DOI:** 10.3390/vaccines10081288

**Published:** 2022-08-10

**Authors:** Angela Bechini, Alfredo Vannacci, Giada Crescioli, Niccolò Lombardi, Marco Del Riccio, Giuseppe Albora, Jonida Shtylla, Marco Masoni, Maria Renza Guelfi, Paolo Bonanni, Sara Boccalini

**Affiliations:** 1 Department of Health Sciences, University of Florence, 50134 Florence, Italy; 2 Department of Neurosciences, Psychology, Drug Research and Child Health, University of Florence, 50134 Florence, Italy; 3 Medical School of Hygiene and Preventive Medicine, University of Florence, 50134 Florence, Italy; 4 SIAF—Digital Learning and IT Training Process Unit, Area for the Innovation and Management of Information and Computer Systems, University of Florence, 50141 Florence, Italy; 5 Department of Experimental and Clinical Medicine, University of Florence, 50134 Florence, Italy

**Keywords:** vaccine hesitancy, behaviour, acceptance, students, medical doctors, pharmacists, training, SARS-CoV-2 vaccine

## Abstract

Background: Healthcare students that refuse to get vaccinated may expose themselves and their patients to several vaccine-preventable diseases, especially during outbreaks or at peak epidemic activity, becoming a threat to themselves and their patients. This study aimed to assess their attitudes towards and perception of vaccines and vaccination. Methods: An anonymous questionnaire was shared with medical students, pharmacy students and medical residents in Hygiene and Preventive Medicine at the University of Florence (Italy), in February 2021. The questionnaire contained 39 questions with open, multi-choice, yes–no, Likert scale answers. A Vaccine Hesitancy Index (VHI) was then calculated. A descriptive statistical analysis was performed. Results: A total of 473 students participated in this study. All students were in favour of vaccination (99.2%) but a relatively low number of participants judged their level of knowledge about vaccinations as “good” (21.8%) or “excellent” (0.6%). About half of students declared that they are not adequately trained during their academic courses. The VHI showed low levels of vaccine hesitancy (mean ± SD 0.38 ± 0.16); moreover, the students were willing to get vaccinated against SARS-CoV-2 when recommended (88.2%) and thought that these vaccines are generally safe. Few students considered the development of SARS-CoV-2 vaccines (13.1%) and the procedures for evaluating clinical trials for marketing authorisation of these vaccines (12.9%) too fast to guarantee their efficacy and safety. Conclusions: Since vaccination and vaccine hesitancy and acceptance topics are being paid increasing attention by the population, new strategies to increase future healthcare professionals’ willingness to promote vaccination and get vaccinated, as well as knowledge on vaccines and vaccination, will be of the utmost importance to fight vaccine preventable diseases.

## 1. Introduction

Globally, infectious diseases are among the most common causes of hospitalisations and death in children before the introduction of vaccines [1]. Since the first moment of its introduction, immunisation has been one of the safest [2], most affordable and viable methods of preventing infectious diseases, and in recent decades, vaccines against deadly diseases such as pneumonia, diphtheria, hepatitis B, measles, polio, pertussis, meningitis, tetanus and rotavirus contributed to sparing many lives [3]. Despite their success (it is estimated that vaccines have prevented 6 million deaths from vaccine-preventable diseases annually [4]) and their reduced cost and availability, a share of the population refuses to get vaccinated (and/or do not want their children to get vaccinated) and is sceptical toward the use of vaccines.

Vaccine hesitancy is defined as a “delay in acceptance or refusal of vaccination despite the availability of vaccination services”, with several factors affecting it (such as the type of vaccine or the microbe against which it is directed), according to the Strategic Advisory Group of Experts on Immunization (SAGE) [5]. As an example, the COVID-19 pandemic caused by SARS-CoV-2 arose suddenly in the early 2020 and new vaccine platforms (e.g., mRNA and vectorial vaccines) were quickly studied in order to control the infective emergency. This fast development process (approximately 11 months) increased the initial hesitancy of the people. It was estimated that COVID-19 vaccine hesitancy varied widely, ranging from 6.4% in Spain to 61.8% in Bulgaria in the adult population [6]. However, in Italy, a study that was published days before the roll-out of the COVID-19 vaccine reported that 9% of the respondents were unwilling to get vaccinated against SARS-CoV-2 [7], which is roughly the same proportion of adults that completed the schedule at the moment [8].

Vaccine hesitancy is even more problematic when it is found in healthcare workers or students in healthcare settings, for three main reasons: these professionals may expose themselves and patients to several vaccine-preventable diseases, especially during outbreaks or at peak epidemic activity, acting as a threat to themselves and their patients. Moreover, hesitant subjects are more prone to absenteeism due to vaccine-preventable diseases [9]. Third, their influence over their patients could affect vaccination uptake, as they usually act as advisors on those questions or misconceptions about vaccination that may be neglected and lead the patient to the choice of not getting vaccinated [10]. This is why the correct training of healthcare professionals and students on vaccines and vaccinations should not be neglected [11].

### Aim of the Study

The main aim of this study was to assess the attitudes and perceptions towards vaccines and the vaccinations of students enrolled in the single-cycle degrees in Medicine and Surgery, Pharmacy, and in the postgraduate school of Hygiene and Preventive Medicine of the University of Florence (Italy). In particular, this evaluation was performed right after the roll-out of COVID-19 vaccines, when the attention of the population on the topic was at its peak.

## 2. Material and Methods

### 2.1. Study Participants

During the period of February–April 2021, an elective teaching activity (ETA) course (16 h) on vaccines for university students in the healthcare setting was organised by the University of Florence. Particularly, the students of III, IV and V year of the Degree Courses in Pharmacy, students of III, IV, V and VI year of the Degree Courses in Medicine and Surgery, and medical residents of the Specialization Medical School in Hygiene and Preventive Medicine (I and II years) at the University of Florence were asked whether they wanted to voluntarily attend the ETA.

The main topics addressed were the different types of available vaccines, the preclinical and clinical development of a vaccine, authorisation process, production, supply, storage, dispensation of vaccines, vaccine vigilance, the Health Technology Assessment (HTA) of new vaccines or vaccination strategies, false myths and scientific truths about vaccinations, vaccination calendars and coverage and, lastly, the impact of vaccination. Many hours of the course were dedicated to the newly available COVID-19 vaccines. In order to assess students’ attitudes and perceptions of vaccines, students were asked to fill in an anonymous questionnaire before the learning activity.

### 2.2. The Anonimus Questionaire

The students could fulfil the questionnaire through the learning management system (LMS) called “Moodle”. The questionnaire contained 39 questions with open, multi-choice, yes/no, Likert scale answers. The questions investigated the compliance with vaccinations and perception of the main topics of the ETA. Each student agreed to participate after receiving brief information about the survey objective. No specific health information was requested from the participants, and, above all, the online questionnaire was entirely anonymous. No information could be traced back to the participants. The collected data do not compromise the students’ privacy. Therefore, ethical approval for the study was not needed. The study was conducted according to the guidelines of the Declaration of Helsinki. Data were collected and managed in aggregated form according to European Union Regulation 2016/679 of the European Parliament and the Italian Legislative Decree 2018/101.

### 2.3. Statistical Analysis

Results obtained through the questionnaire were analysed. A descriptive statistical analysis was performed. Data were tested for normality using the Shapiro–Wilk test and for homogeneity through the Cochran’s test. Categorical data were reported as numbers and percentages and compared through the Chi-square test, while continuous data were reported as mean and standard deviation (SD) or median and interquartile range (IQR), and compared with the Student’s t-test or the Wilcoxon’s test, respectively. Results are considered statistically significant with a *p*-value of 0.05.

### 2.4. Vaccine Hesitancy Index

On the basis of previous publications [12], a “Vaccine Hesitancy Index” (VHI) was calculated for each student. In particular, question 5 included eight Likert-type statements to which the participants were asked to declare their agreement or disagreement. The statements were the following: (A1) Vaccines are important for human health; (A2) Vaccines are effective; (A3) Vaccines are safe; (A4) Vaccines are in line with my ideas; (B1) Vaccines are dangerous for human health; (B2) Vaccines are not adequately studied in clinical trials; (B3) Vaccines are not adequately controlled during their production processes; (B4) Vaccines are produced and recommended only for the economic interest of pharmaceutical companies.

The level of agreement or disagreement was scored as follows: “totally agree” = 1; “partially agree” = 2; “partially disagree” = 3; and “totally disagree” = 4. Thus, for the first four statements (A1–A4), the higher the score, the lower the propensity towards vaccines, while for the second four (B1–B4), the higher the score, the higher the propensity. The VHI was calculated as follows: VHI = [(A1 + A2 + A3 + A4)/4]/[(B1 + B2 + B3 + B4)/4].

Therefore, the VHI ranged between a minimum of 0.25 and a maximum of 4. A higher value of VHI showed high hesitancy towards vaccinations.

## 3. Results

The questionnaire was administered to a total of 473 students, among which 186 (39.3%) were enrolled on the single-cycle degree in Pharmacy, 263 (55.6%) were enrolled on the single-cycle degree in Medicine, and 24 (5.1%) were enrolled on the postgraduate school of Hygiene and Preventive Medicine. The majority of students were female (*n* = 321, 67.9%), and in their IV (*n* = 67, 14.2%) or V (*n* = 358, 75.7%) academic year (Table 1).

Overall, all students were in favour of vaccines and vaccinations (*n* = 469, 99.2%) without statistically significant differences among the different courses. A relatively low number of participants judged their level of knowledge about vaccines and vaccinations as “good” (*n* = 103, 21.8%) or “excellent” (*n* = 3, 0.6%), with statistically significant differences among the three courses with the highest positive values of personal evaluation among Medicine students or postgraduates (*p* < 0.001). Information on vaccines and vaccinations was mostly retrieved from academic courses (*n* = 423, 89.4%), books and scientific journals (*n* = 283, 59.8%), institutional websites (*n* = 259, 54.8%), and traditional mass media (*n* = 164, 34.7%). Of notice, the majority of Pharmacy (*n* = 110, 59.1%) and Medicine (*n* = 119, 42.3%) students declared that they were not adequately trained and informed on the subject of vaccines and vaccinations during their academic courses (*p* < 0.001) (Table 2).

**Figure 1:**
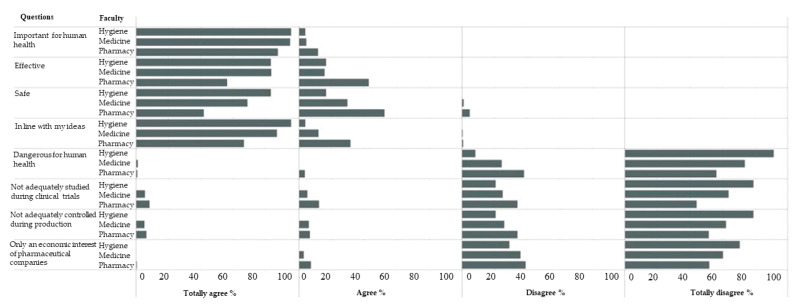
The percentage of students who agreed or disagreed with the eight Likert-type statements.

The calculation of the VHI showed that vaccine hesitancy was relatively low in all student groups considered (mean ± SD 0.38 ± 0.16), resulting slightly higher for Pharmacy students (mean ± SD 0.44 ± 0.18) (*p* < 0.001) (Table 3). The percentages of students who agreed or disagreed with the eight Likert-type statements proposed in Question 5 are reported in Figure 1.

Considering students’ attitudes and perceptions towards vaccines against SARS-CoV-2, most of them were in favour of getting vaccinated (*n* = 417, 88.2%) (*p* = 0.001), and they thought that both mRNA (*n* = 401, 84.8%) and viral vector (*n* = 362, 76.5%) vaccines are safe (*p* < 0.001). Furthermore, few students have considered the development of SARS-CoV-2 vaccines (*n* = 62, 13.1%) and the procedures for evaluating clinical trials for marketing the authorisation of these vaccines (*n* = 61, 12.9%) which are too fast to guarantee their efficacy and safety (*p* < 0.001). Noteworthy, for all questions concerning SARS-CoV-2 vaccines, a non-negligible number of participants did not know how to respond, especially for questions regarding the efficacy and safety of SARS-CoV-2 vaccines (*p* < 0.001). As highlighted by the results of the Chi-square test, statistically significant differences were found in the attitude and perceptions towards COVID-19 vaccines among the students attending the three courses (Table 4).

Considering students’ perceptions regarding the content and composition of vaccines, most of them thought that vaccines contain microorganisms adequately treated to make them harmless and unable to cause disease (*n* = 373, 78.9%) (*p* = 0.038), and students also thought that vaccines do not contain harmful components (*n* = 352, 74.4%). Furthermore, most students considered the number and the amount of adjuvants present in some vaccines not dangerous (*n* = 320, 67.7%). Additionally, in this case, a non-negligible number of participants did not know how to respond, especially to questions regarding the presence of harmful components, including the adjuvants (*p* = 0.001) (Table 5).

Considering students’ perceptions regarding vaccination recommendations, most of them are in favour to continue to vaccinate for diseases that have disappeared or are infrequent in Italy (*n* = 350, 74%) (*p* = 0.001). Overall, students do not think that in Italy there are too many recommended paediatric vaccinations (*n* = 424, 89.6%), and they agree with the decision to introduce compulsory vaccinations for school attendance (*n* = 418, 88.4%). Epidemiological evaluations (*n* = 320, 67.7%) and disease impact assessments (*n* = 315, 66.6%) are considered the best criteria to recommend the introduction of a new vaccine in the vaccination plan, followed by vaccine efficacy and safety assessments. Particularly, all reported options (that are the domains of the HTA applied to vaccines and vaccinations) are considered the fundamental criteria to follow in the decision process of new recommendations by approximately 32% of students (*n* = 152). It is noteworthy that a high number of participants did not know how to respond, especially to questions regarding vaccines and vaccinations during pregnancy (*p* < 0.001) (Table 6).

Students’ perceptions regarding the testing, production, storage and dispensing of vaccines and the pharmacovigilance of vaccines are reported in Supplementary Tables S1 and S2.

## 4. Discussion

We assessed the attitudes and perceptions towards vaccines and vaccinations of students enrolled on the single-cycle degrees in Medicine and Surgery, Pharmacy, and in the postgraduate school of Hygiene and Preventive Medicine at the University of Florence (Italy). The evaluation was performed when the incidence of COVID-19 was high, COVID-19 vaccination had just begun for high-risk groups and new data or alarms were daily reported on media in Italy [13].

The evaluation of the attitudes and perceptions towards vaccines and vaccinations in the participants in our study is particularly important as they will represent tomorrow’s healthcare workers, and most of them will have a leading role to play in addressing the emergence of vaccine hesitancy among patients and fostering vaccine acceptance (future general practitioners, paediatricians, public health specialists, etc.) [14]. These assessments should indeed be promoted and realised routinely, to both meet the educational needs of the student population and set up strategies aimed at increasing vaccine acceptance and critical reasoning towards fake news and sources chosen to retrieve information about vaccines and vaccinations. In our sample, students reported the high utilisation of books and scientific journals and institutional websites (more than traditional mass media), and most of them stated that academic courses were the most important resource to obtain information on vaccines. This was also observed by Qiao et al. [15], who reported health agencies as the main sources of information among a sample of university students, and by Gallé et al., who identified healthcare personnel/scientists as the main source of information on vaccines and vaccinations among a sample of undergraduate students [16]. The situation is different, compared to the general population, where a bigger and growing role is played by the Internet and social media, especially during the COVID-19 pandemic and the roll-out of COVID-19 vaccines [17,18]. A recent institutional survey reported how four out of five web users in Italy used the Internet to find health information [19].

Another important result that needs to be highlighted is that the students in our sample recognise the need to vaccinating for diseases that have apparently disappeared in Italy, understanding the importance of prevention and the possibility that some infectious diseases may return even if were deemed disappeared [20]. Moreover, this study found high vaccine acceptance among students, who considered vaccines safe, adequately produced and free of harmful components; overall, the students reported relatively low levels of vaccine hesitancy. This is not surprising, considering that the study was conducted among students of healthcare settings that are trained in the field of vaccines and vaccinations: results reported by studies that were recently published in the literature are consistent with this finding [21]. However, it should also be highlighted that a non-negligible share of the participants (more than half for Pharmacy students) declared that they were not adequately trained and informed about vaccines and vaccinations during their academic course, despite reporting high scores on vaccine knowledge in general, in the pre-assessment test that was conducted before the elective activity [11]. Future healthcare providers need to have a good knowledge of vaccines (risks, recommendations) and also be confident when they have a conversation with their patients. The time spent learning about immunisation was in fact shown to be associated with confidence in answering patients’ questions [22].

This study has a number of limitations. Results obtained by interviewing healthcare students are hardly generalisable, as they represent a specific group with high literacy levels and specific training on vaccines and vaccinations. Moreover, the students that participated in the elective activity may be those with the biggest interest towards vaccines and vaccinations, and therefore those who are more inclined to accept and promote vaccinations. Furthermore, it was not possible to enrich the study with more demographic data such as age, which would give a more in-depth understanding of the presented phenomenon. Finally, the information collected mainly referred to vaccines in general, but more in-depth information should be analysed to understand whether there are specific differences between the different types of vaccines (e.g., COVID-19).

Despite its limitations, to our knowledge, this is the first study that assessed attitudes and perceptions towards the vaccines and vaccinations of healthcare students in Italy, and it can provide valuable information for further research that will use different designs (e.g., a survey administered to a nationwide representative sample of students) to deepen this topic. Furthermore, the use of VHI made the estimation of the phenomenon of vaccine hesitancy simple and effective. Although this is a small and selected sample of subjects who have received specific training on vaccines, the VHI represents a suitable tool for the analysis of vaccine hesitancy even in much larger and heterogeneous samples.

## 5. Conclusions

Healthcare students in our sample have been proven to have an overall positive attitude towards vaccinations; as future healthcare workers, they will become crucial resources in spreading essential and correct information to the general public. Enthusiastic and literate students will be important to promote vaccinations in order to increase vaccination rates in the future and increase public trust not only in vaccinations but also in medical professionals and the healthcare system by creating innovative campaigns and using new forms of communication. However, it is essential that healthcare students are adequately and thoroughly trained on vaccines and vaccinations during their educational courses in the future through specifically structured lessons and not in ad hoc activities. Particularly attention to training should be paid in non-medical healthcare academic courses.

## Figures and Tables

**Table 1:** Characteristics of the sample (*n* = 473 students).

	Year of Study				Gender	
Faculty	III *n* (% in Row)	IV *n* (% in Row)	V or More *n* (% in Row)	Postgraduate *n* (% in Row)	Males *n* (% in Row)	Females *n* (% in Row)
Pharmacy *n* = 186	15 (8.1)	54 (29.0)	117 (62.9)	-	41 (22.0)	145 (78.0)
Medicine *n* = 263	9 (3.4)	13 (4.9)	241 (91.6)	-	103 (39.2)	160 (60.8)
Hygiene *n* = 24	-	-	-	24	8 (33.3)	16 (66.7)
Total	24 (5.1)	67 (14.2)	358 (75.7)	24 (5.1)	152 (32.1)	321 (67.9)

**Table 2:** General questions about the knowledge on vaccines and sources of information on this topic.

Question and Answers	Pharmacy *n* = 186 (%)	Medicine *n* = 263 (%)	Hygiene *n* = 24 (%)	*p-*Value
**In general, are you in favour of vaccinations?**				
Yes	183 (98.4)	262 (99.6)	24	0.335
No	-	-	-	
I don’t know	3 (1.6)	1 (0.4)	-	
**How do you rate your current level of knowledge about vaccines**				
Absent	-	1 (0.4)	-	<0.001
Scarce	37 (19.9)	30 (11.4)	2 (8.3)	
Insufficient	35 (18.8)	37 (14.1)	3 (12.5)	
Sufficient	89 (47.9)	127 (48.3)	6 (25.0)	
Good	23 (12.4)	68 (25.9)	12 (50.0)	
Excellent	2 (1.1)	-	1 (4.2)	
**To date, where/from whom did you get information on vaccinations? (it is possible to indicate more than one answer)**				
Word of mouth (family, friends)	52 (28.0)	78 (29.7)	5 (20.8)	
Books, scientific journals	80 (43.0)	184 (70.0)	19 (79.2)	
Institutional websites	73 (39.3)	170 (64.6)	16 (66.7)	
Blog/Forum/Non-institutional sites	24 (12.9)	46 (17.5)	2 (8.3)	
Traditional mass media (TV, radio, newspapers)	61 (32.8)	102 (38.8)	1 (4.2)	
Vaccination service doctor	11 (5.9)	42 (16.0)	5 (20.8)	
Family doctor (General practitioner)	50 (26.9)	85 (32.3)	5 (20.8)	
Pediatrician	18 (9.7)	30 (11.4)	1 (4.2)	
Private medical practitioner	10 (5.4)	21 (8.0)	1 (4.2)	
Gynecologist, obstetrician, clinic	5 (2.7)	13 (4.9)	-	
School	35 (18.8)	64 (24.3)	2 (8.3)	
University	158 (85.0)	242 (92.0)	23 (95.8)	
Other *	2 (1.1)	6 (2.3)	1 (4.2)	
**Do you think that the student attending the degree of Medicine and Surgery/Pharmacy is adequately trained and informed on the subject of vaccinations during his study course?**				
Yes	41 (22.0)	93 (35.4)	3 (12.5)	<0.001
No	110 (59.1)	119 (42.3)	-	
I don’t know	35 (18.8)	51 (19.4)	21 (87.5)	

* 5 students reported asking information to a hygienist, 2 to a cardiologist, 1 to a surgeon, 1 to an internist, 1 to an emergency clinician, 1 to a nephrologist, 1 to a neurologist, 1 to an immunologist and 1 to a homeopath.

**Table 3:** Vaccine Hesitancy Index.

VHI	Overall	Pharmacy *n* = 186 (%)	Medicine *n* = 263 (%)	Hygiene *n* = 24 (%)	*p*-Value *
Mean ± SD	0.38 ± 0.16	0.44 ± 0.18	0.34 ± 0.14	0.30 ± 0.09	<0.001
Median (IQR)	0.31 (0.25–0.46)	0.40 (0.28–0.54)	0.29 (0.25–0.4)	0.25 (0.25–0.32)	

IQR: interquartile range; SD: standard deviation; VHI: vaccine hesitancy index. * Test di Kruskall—Wallis.

**Table 4:** Attitudes and perceptions towards vaccines against SARS-CoV-2.

Questions and Answers	Pharmacy *n* = 186 (%)	Medicine *n* = 263 (%)	Hygiene *n* = 24 (%)	*p*-Value
**Are you in favour of getting vaccinated against SARS-CoV-2?**				
Yes	149 (80.1)	246 (93.5)	22 (91.7)	0.001
No	2 (1.1)	4 (1.5)	-	
I don’t know	9 (4.5)	2 (0.8)	-	
It depends on the type of vaccine I am offered	26 (14.0)	10 (3.8)	2 (8.3)	
No answer	-	1 (0.4)	-	
**Do you think mRNA vaccines for the prevention of disease caused by SARS-CoV-2 disease, as they are made, are safe**?				
Yes	138 (74.2)	242 (92.0)	21 (87.5)	<0.001
No	6 (3.2)	1 (0.4)	-	
I don’t know	42 (22.6)	19 (7.2)	3 (12.5)	
No answer	-	1 (0.4)	-	
**Do you think that the viral vector vaccines for the prevention of disease caused by SARS-CoV-2, as they are made, are safe?**				
Yes	113 (60.8)	227 (86.3)	22 (91.7)	<0.001
No	4 (2.2)	-	-	
I don’t know	69 (37.1)	36 (13.7)	2 (8.3)	
**Do you think that the development of the SARS-CoV-2 vaccines already on the market has been too fast to the detriment of their safety and/or their effectiveness?**				
Yes	42 (22.6)	19 (7.2)	1 (4.2)	<0.001
No	101 (54.3)	193 (73.4)	18 (75.0)	
I don’t know	43 (23.1)	51 (19.4)	5 (20.8)	
**Do you think that the procedure for evaluating clinical trials for marketing authorization by the regulatory agencies for the now available SARS-CoV-2 vaccines was too fast to the detriment of their safety and/or efficacy?**				
Yes	35 (18.8)	24 (9.1)	2 (8.3)	0.008
No	101 (54.3)	182 (69.2)	17 (70.8)	
I don’t know	50 (26.9)	57 (21.7)	5 (20.8)	

**Table 5:** Perceptions regarding the content and composition of vaccines.

Questions and Answers	Pharmacy *n* = 186 (%)	Medicine *n* = 263 (%)	Hygiene *n* = 24 (%)	*p*-Value
**Do you think that the vaccine vials may contain microorganisms (from which the vaccines are obtained) not adequately treated to make them harmless and unable to cause disease?**				
Yes	18 (9.7)	18 (6.8)	-	0.038
No	134 (72.0)	218 (82. 9)	21 (87.5)	
I don’t know	34 (18.3)	27 (10.3)	3 (12.5)	
**Do you think vaccine vials may contain harmful components?**				
Yes	13 (7.0)	26 (9.9)	-	0.213
No	135 (72.6)	194 (73.8)	23 (95.8)	
I don’t know	37 (19.9)	42 (16.0)	1 (4.2)	
No answer	1 (0.5)	1 (0.4)	-	
**Do you think the amount of adjuvants (e.g., aluminium salts) present in some vaccines is dangerous?**				
Yes	7 (3.8)	5 (1.9)	-	0.001
No	107 (57.5)	192 (73.0)	21 (87.5)	
I don’t know	63 (33.9)	64 (24.3)	3 (12.5)	
I don’t know what an adjuvant is	9 (4.8)	2 (0.8)	-	
**Why do you think the amount of adjuvants in some vaccines is dangerous?**				
No answer	179 (96.2)	257 (97.7)	24 (100)	0.797
Adjuvants are harmful substances	2 (1.1)	1 (0.4)	-	
The amount of adjuvants present in some vaccines are excessive	5 (2.7)	4 (1.5)	-	
Other	-	1 (0.4)	-	

**Table 6:** Perceptions regarding vaccination recommendations.

Questions and Answers	Pharmacy *n* = 186 (%)	Medicine *n* = 263 (%)	Hygiene *n* = 24 (%)	*p*-Value
**In your opinion, today we must continue to vaccinate for diseases that have disappeared or are infrequent in Italy, such as polio and diphtheria?**				
Yes	123 (66.1)	203 (77.2)	24	0.001
No	27 (14.5)	16 (6.1)	-	
I don’t know	36 (19.4)	44 (16.7)	-	
**In your opinion, is the measles vaccine dangerous because it can cause autism?**				
Yes	1 (0.5)	-	-	<0.001
No	6 (3.2)	2 (0.8)	-	
I don’t know	153 (82.3)	251 (95.4)	24	
No answer	1 (0.5)	-	-	
**In your opinion, are too many paediatric vaccinations recommended today?**				
Yes	12 (6.5)	7 (2.7)	-	0.137
No	162 (87.1)	238 (90.5)	24	
I don’t know	12 (6.5)	18 (6.8)	-	
**Do you agree with the decision to introduce compulsory vaccinations for school attendance?**				
Yes	163 (87.6)	235 (89.4)	20 (83.3)	0.800
No	10 (5.4)	16 (6.1)	2 (8.3)	
I don’t know	12 (6.5)	12 (4.6)	2 (8.3)	
No answer	1 (0.5)	-	-	
**Would you recommend any vaccinations to a pregnant woman?**				
Yes	46 (24.7)	160 (60.8)	22 (91.7)	<0.001
No	45 (24.2)	13 (4.9)	-	
I don’t know	94 (50.5)	89 (33.8)	2 (8.3)	
No answer	1 (0.5)	1 (0.4)	-	
**In your opinion, based on what criteria should be decided whether to recommend a new vaccine in the vaccination plan? (it is possible to indicate more than one answer)**				
Epidemiological evaluations	141 (75.8)	168 (63.9)	11 (45.8)	
Disease Impact assessments	138 (74.2)	166 (63.1)	11 (45.8)	
Vaccine efficacy and safety assessments	119 (64.0)	146 (55.5)	10 (41.7)	
Evaluation of possible alternatives	34 (18.3)	56 (21.3)	5 (20.8)	
Economic assessments	20 (10.8)	59 (22.4)	5 (20.8)	
Organisational evaluations	21 (11.3)	52 (19.8)	5 (20.8)	
Ethical-social evaluations	32 (17.2)	51 (19.4)	2 (8.3)	
All previous answers	31 (16.7)	106 (40.3)	15 (62.5)	

## Data Availability

Data supporting reported results are available upon request to the corresponding author. Data were collected and managed in aggregated form according to European Union Regulation 2016/679 of European Parliament and the Italian Legislative Decree 2018/101.

## Supplementary Materials

The following supporting information can be downloaded at: https://www.mdpi.com/article/10.3390/vaccines10081288/s1; Supplementary Table S1. Perceptions regarding the testing, production, storage and dispensing of vaccines: the influence of these processes on efficacy and safety; Supplementary Table S2. Perceptions regarding the pharmacovigilance of vaccines.
