# Supplementary material for: Attitudes and Perceptions of University Students in Healthcare Settings towards Vaccines and Vaccinations Strategies during the COVID-19 Pandemic Period in Italy
Source: Vaccines (Basel). 2022 Aug 10;10(8):1288. doi: 10.3390/vaccines10081288 (PMC9413643; doi:10.3390/vaccines10081288)
Supplement: Supplementary file 1 [file vaccines-10-01288-s001.zip › vaccines-1859790-supplementary.pdf]

Supplementary Tables

Supplementary Table S1. Perceptions regarding the testing, production, storage and dispensing of vaccines: the influence of these processes on efficacy and safety

**Questions and answers**	**Pharmacy N = 186 (%)**	**Medicine N = 263 (%)**	**Hygiene N = 24 (%)**	***p-value***
**In general, do you think that the studies carried out during the clinical development of a vaccine are sufficient to determine its efficacy?**				
Yes	133 (71.5)	200 (76.1)	17 (70.8)	0.294
No	25 (13.4)	19 (7.2)	3 (12.5)	
Don't know	28 (15.1)	44 (16.7)	4 (16.7)	
**In general, do you think that the studies carried out during the clinical development of a vaccine are sufficient to determine its safety?**				
Yes	113 (60.8)	189 (71.9)	21 (87.5)	0.001
No	39 (21.0)	24 (9.1)	1 (4.2)	
Don't know	34 (18.3)	50 (19.0)	2 (8.3)	
**In your opinion, compared to drugs, the production of vaccines is**				
More subjected to quality controls	45 (24.2)	80 (30.4)	9 (37.5)	0.276
Less subjected to quality controls	10 (5.4)	8 (3.0)	-	
Equally subjected to quality controls	131 (70.4)	175 (66.5)	15 (62.5)	
**Do you think there are adequate controls during the manufacturing process to ensure that the vaccine is not contaminated with impurities?**				
Yes	144 (77.4)	223 (84.8)	23 (95.8)	0.024
No	6 (3.2)	1 (0.4)	-	
Don't know	36 (19.4)	39 (14.8)	1 (4.2)	
**Do you think that the supply and storage of vaccines is always carried out in such a way as to ensure their efficacy and safety?**				
Yes	113 (60.8)	145 (55.1)	16 (66.7)	0.683
No	19 (10.2)	23 (8.8)	2 (8.3)	
Don't know	54 (29.0)	94 (35.7)	6 (25.0)	
No answer	-	1 (0.4)	-	
**Do you think there are adequate controls during the vaccine supply and storage process?**				
Yes	117 (62.9)	148 (56.3)	17 (70.8)	0.593
No	8 (4.3)	13 (4.9)	-	
Don't know	61 (32.8)	101 (38.4)	7 (29.2)	
No answer	-	1 (0.4)	-	
**Do you believe that vaccine dispensing procedures may affect their efficacy and safety?**				
Yes	118 (63.4)	168 (63.9)	18 (75.0)	0.184
No	43 (23.1)	44 (16.7)	4 (16.7)	
Don't know	25 (13.4)	51 (19.4)	2 (8.3)	
**Do you think that the dispensing of vaccines by qualified and competent professionals could affect their correct use?**				
Yes	114 (61.3)	180 (68.4)	18 (75.0)	0.392
No	46 (24.7)	54 (20.5)	5 (20.8)	
Don't know	26 (14.0)	29 (11.0)	1 (4.2)	

Supplementary Table S2. Perceptions regarding the pharmacovigilance of vaccines

Questions and answers	Pharmacy N = 186 (%)	Medicine N = 263 (%)	Hygiene N = 24 (%)	*p-value*
**Do you think vaccines are adequately monitored by pharmacovigilance?**				
Yes	145 (78.0)	226 (85.9)	22 (91.7)	0.065
No	11 (5.9)	5 (1.9)	1 (4.2)	
Don't know	30 (16.1)	32 (12.2)	1 (4.2)	
**In your opinion, does the pharmacovigilance on vaccines guarantee their safety?**				
Yes	129 (69.4)	206 (78.3)	20 (83.3)	0.123
No	19 (10.2)	16 (6.1)	-	
Don't know	38 (20.4)	41 (15.6)	4 (16.7)	
**In your opinion, the precautionary withdrawal of some batches of a vaccine indicates that**				
Controls in production are ineffective and insufficient	24 (12.9)	18 (6.8)	1 (4.2)	0.118
The control mechanism is so effective that suspicious batches are immediately withdrawn	130 (69.9)	202 (76.8)	21 (87.5)	
Don't know	32 (17.2)	43 (16.4)	2 (8.3)	
**In your opinion, are the procedures and typical problems of adverse reactions management in pharmacovigilance the same for drugs and vaccines?**				
Yes	51 (27.4)	124 (47.1)	10 (41.7)	<0.001
No	92 (49.5)	71 (27.0)	7 (29.2)	
Don't know	43 (23.1)	68 (25.9)	7 (29.2)	
